# Uremic serum damages endothelium by provoking excessive neutrophil extracellular trap formation

**DOI:** 10.1038/s41598-021-00863-w

**Published:** 2021-11-02

**Authors:** Hoi Woul Lee, Victor Nizet, Jung Nam An, Hyung Seok Lee, Young Rim Song, Sung Gyun Kim, Jwa-Kyung Kim

**Affiliations:** 1 Department of Clinical Immunology, Hallym University Sacred Heart Hospital, Anyang, South Korea; 2 Division of Host-Microbe Systems and Therapeutics, Department of Pediatrics, UC San Diego, La Jolla, CA USA; 3 Department of Internal Medicine & Kidney Research Institute, Hallym University Sacred Heart Hospital, Hallym University College of Medicine, Pyungan-dong, Dongan-gu, Anyang, 431-070 South Korea

**Keywords:** Immunology, Nephrology

## Abstract

Cardiovascular disease (CVD) is the leading cause of death in patients with chronic kidney disease (CKD). Endothelial cell (EC) dysfunction is a key CKD-specific risk factor; however, the mechanisms by which uremia harms the endothelium are still unclear. We report a role for excessive neutrophil extracellular trap (NET) formation induced by uremic serum on EC injury. Level of plasma nucleosome and myeloperoxidase-DNA, established in vivo markers of NETs, as well as intracellular adhesion molecule (ICAM)-1 were measured in hemodialysis (HD) patients and healthy volunteers (HV) and their prognostic role evaluated. For in vitro studies, HV-derived neutrophils and differentiated HL-60 cells by retinoic acid were used to determine the effect of uremic serum-induced NETs on human umbilical vein EC (HUVEC). The level of in vivo NETs was significantly higher in incident HD patients compared to HV, and these markers were strongly associated with ICAM-1. Specifically, nucleosome and ICAM-1 levels were independent predictors of a composite endpoint, all-cause mortality, or vascular access failure. In vitro, HD-derived uremic serum significantly increased NET formation both in dHL-60 and isolated neutrophils compared to control serum, and these NETs decreased EC viability and induced their apoptosis. In addition, the level of ICAM-1, E-selectin and von Willebrand factor in HUVEC supernatant was significantly increased by uremic serum-induced NETs compared to control serum-induced NETs. Dysregulated neutrophil activities in the uremic milieu may play a key role in vascular inflammatory responses. The high mortality and CVD rates in ESRD may be explained in part by excessive NET formation leading to EC damage and dysfunction.

## Introduction

Chronic kidney disease (CKD) is a global problem with high morbidity and mortality. Patients with CKD have a high prevalence of cardiovascular disease (CVD) and associate risk factors such as hypertension, diabetes, old age, and the metabolic syndrome^1–3^. However, these risk factors cannot fully explain the exceptionally high CVD rates in patients with CKD. Therefore, possible contributions of other factors, such as endothelial dysfunction, have been studied^4,5^.

Maintaining the functional integrity of endothelium is important for prevention of vascular diseases. As renal function declines, various uremic toxins and metabolic abnormalities can induce damaging effects on host cells and tissues, also known as uremic toxicity. These uremic toxins may be potentially a non-traditional, CKD-specific CV risk factor^6–8^. When renal dysfunction progresses to end-stage renal disease (ESRD), chronic retention of these uremic substances can completely alter the internal environment known as uremic milieu, which is characterized by chronic low-grade inflammation and increased oxidative stress. This uremia-associated low-grade inflammation in the vascular wall is an important contributor to the pathophysiology of endothelial cell (EC) damage and dysfunction^8–10^.

Endothelial dysfunction initiates vascular remodeling and can be an early determinant of accelerated atherosclerosis^11,12^. Ischemic heart disease as well non-obstructive vascular diseases (such as increased vascular stiffness, calcification, and ossification) are highly prevalent in CKD patients^13^. Endothelial dysfunction begins early in the progression of CKD, and results from an imbalance between EC damage and repair^8,14,15^. Most uremic toxins are protein-bound compounds that are poorly removed by hemodialysis (HD)^16,17^. Therefore, endothelial dysfunction and associated vascular complications can progress even after HD. However, the exact mechanisms by which uremia or the uremic milieu influences EC dysfunction, and especially the early responses of EC injury, are unclear.

Neutrophils play a crucial role in immune defense against pathogens. In addition to classical phagocytosis, neutrophils are actively involved in early stage atherosclerosis and amplify local inflammation and tissue damage by recruiting other immune cells^18^. In one potential outcome of neutrophil activation, the cells can release chromatin, nuclear proteins, and serine proteases extracellularly in a specialized cell death process known as neutrophil extracellular trap (NET) formation^19–21^. Besides their direct antimicrobial activity, excessive NET formation has important roles in sterile inflammation, such as autoimmune and metabolic diseases, and vascular inflammation^20,22^. Indeed, neutrophils attach themselves to atherosclerotic plaques primarily through NET formation, such that NETs themselves are source of peptides deposited directly on the inflamed endothelial surface in atherosclerotic vessels^23,24^. In this manner, NETs directly interact with ECs, resulting in endothelial dysfunction^25,26^.

Nucleosomes, sections of chromosomal DNA wrapped around a core of proteins, are an established in vivo maker of NETosis. Circulating nucleosomes are present at significantly higher levels in incident ESRD patients compared to controls and so could be a prognostic marker of long-term mortality^27^. Since NETosis is a complex process that could trigger EC activation and inflammation, we tested the hypothesis that the increased NETs in a uremic milieu provoke vascular EC damage and injury. Our findings unveil a novel mechanism by which increased NETosis in the uremic milieu of CKD induces endothelial dysfunction and subsequent atherosclerosis.

## Results

### In vivo relationship of NETs and ICAM-1 with vascular outcomes

**Figure 1:**
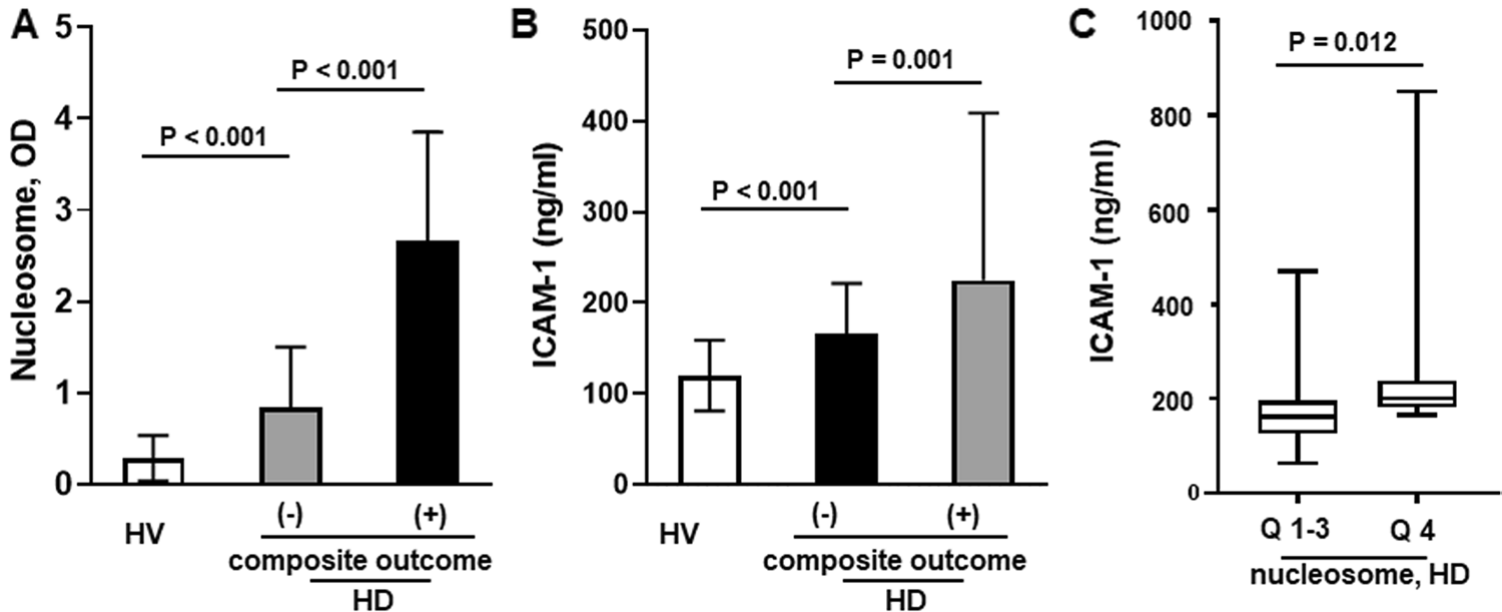
(**A**,**B**) Mean plasma levels of nucleosome and ICAM-1 in HVs, HD patients with the composite outcome, and HD patients without the composite outcome, and the between-group differences. (**C**) Mean ICAM-1 level according to nucleosome level Q1-3 and Q4 in HD patients (independent sample t-test). Patients with a higher nucleosome level had a significantly higher ICAM-1 level.

A total of 201 new HD patients were analyzed in this study. Their mean age was 66.6 ± 11.8 years and the most common cause of ESRD was diabetes (67.7%). During a mean 3-year follow-up, 43 patients died and 42 cases of vascular access failure were observed. The primary composite endpoint, all-cause mortality or loss of vascular access, occurred in 66 cases (32.6%). Baseline clinical and laboratory characteristics were compared between patients with and without composite outcomes and presented in Table 1. Patients with the composite outcome were significantly older and tended to have a higher prevalence of previous CVD than those without. Interestingly, the numbers of peripheral WBCs and neutrophils, the neutrophil/lymphocyte ratio and the hs-C-reactive protein (hsCRP) level were significantly higher in the patients who met the composite endpoint compared to those who did not. In addition, circulating nucleosomes, MPO-DNA, and ICAM-1 levels were significantly higher in patients included in the composite endpoint compared to those not included (Fig. 1A,B).

**Table 1:** Baseline clinical and biochemical data according to the occurrence of composite outcomes.

Variable	Total (n = 202)	Composite outcomes		p
		( +) (n = 66)	(−) (n = 136)	
Age (years)	66.6 ± 11.8	70.4 ± 9.6	64.8 ± 12.2	0.002
Gender, male, n (%)	123 (60.9)	45 (68.2)	78 (57.4)	0.092
SBP (mmHg)	143.3 ± 19.3	146.3 ± 20.8	142.0 ± 20.5	0.133
DBP (mmHg)	77.4 ± 9.2	78.0 ± 12.1	77.1 ± 11.4	0.644
BMI (kg/m^2^)	24.6 ± 5.0	24.4 ± 4.9	24.8 ± 5.1	0.631
Previous cardiovascular events, n (%)	46 (22.7)	20 (30.3)	26 (19.1)	0.063
DM, n (%)	136 (67.7)	46 (69.6)	91 (66.9)	0.436
**Laboratory parameters**				
WBC	6090 ± 1918	7530 ± 1970	6614 ± 1826	0.002
Neutrophil count	4777 ± 1727	5554 ± 1774	4406 ± 1580	< 0.001
Hemoglobin (g/dL)	9.21 ± 1.79	9.10 ± 2.00	9.26 ± 1.68	0.528
Neutrophil/lymphocyte ratio	4.3 ± 2.9	5.3 ± 3.1	3.9 ± 2.4	0.002
Blood urea nitrogen (mg/dL)	82.5 ± 31.2	79.5 ± 26.3	84.4 ± 32.3	0.102
Creatinine (mg/dL)	7.61 ± 3.14	7.91 ± 3.29	7.00 ± 2.67	0.047
Albumin (g/dL)	3.64 ± 0.51	3.55 ± 0.52	3.63 ± 0.51	0.458
Total cholesterol (mg/dL)	153.4 ± 37.9	156.1 ± 41.2	146.0 ± 25.7	0.312
hs-CRP	2.06 ± 1.86	2.93 ± 2.39	1.64 ± 1.55	0.005
**NETosis marker**				
Nucleosome*, median (ranges)	1.20 (0.42–2.83)	2.59 (0.85–3.21)	0.83 (0.32–1.88)	< 0.001
MPO*	0.07 (0.02–0.24)	0.05 (0.02–0.32)	0.04 (0.03–0.23)	0.042
ICAM-1	185.5 ± 117.8	225.6 ± 184.5	166.1 ± 55.7	0.001

Data are expressed as means ± SD except for those with *, which are medians with range.

The circulating nucleosome levels showed a positive correlation with the MPO-DNA level (r = 0.316, p < 0.001) (Supp Fig. 1). As shown in Fig. 1C, the mean ICAM-1 level was significantly increased in the highest nucleosome group, Q4, compared to the Q1–3 groups (201.8 ± 132.4 vs. 179.4 ± 111.8, p = 0.012) suggesting a close association between increased NETosis and endothelial damage. To support this finding, we additionally measured plasma E-selectin and vWF in HV and HD, and similar findings were also observed: plasma E-selectin and vWF was significantly higher in HD patients compared to HV (25,690 ± 16,881 vs. 14,605 ± 9378 with E-selectin, 14,233 ± 9263 vs. 5245 ± 4378 with vWF) (Supp Fig. 2A) and , HD patients in the Q4 group showed significantly increased plasma E-selectin level compared to those in Q1–3 groups (p = 0.014) (Supp Fig. 2B).

### Predictors

**Figure 2:**
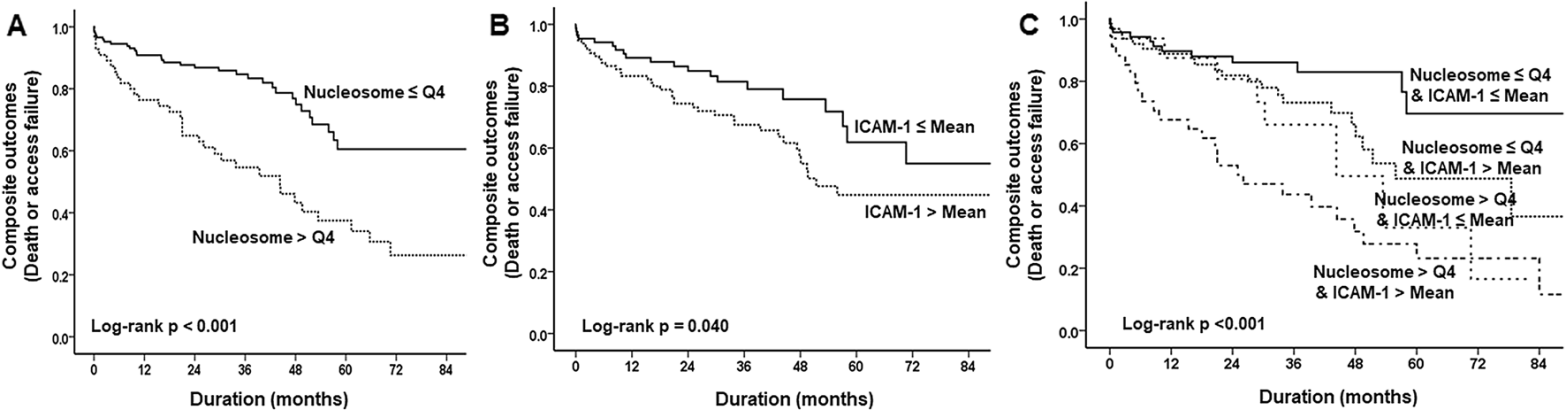
Survival analysis based on nucleosome and ICAM-1 levels. Kaplan–Meier survival curves based on nucleosome level of Q4 and Q1-3, and mean ICAM-1 level (**A**,**B**). A significant difference was observed between the two curves. Patients in nucleosome Q4 and a high ICAM-1 level showed the worst prognosis (**C**).

In the total population, the overall event-free survival (EFS) rates were 87.1%, 77.7%, and 67.3% at 1, 3, and 5 years, respectively. However, for the patients with increased nucleosome levels over the highest quartile, Q4, the survival rates were 76.4%, 56.4%, and 40.0% at 1, 3, and 5 years, respectively. Indeed, an increased circulating nucleosome level (Q4) was a significant adverse prognostic factor for long-term outcomes in incident HD patients (p < 0.001, Fig. 2A). Similarly, a higher mean ICAM-1 level was a major predictor of adverse outcomes (p = 0.040, Fig. 2B). When the two parameters were considered together, patients in the nucleosome Q4 and higher ICAM-1 group showed the lowest 5-year EFS rate (32.4%; p < 0.001, Fig. 2C). On multivariate analysis, (Table 2) age over 65 years, a high nucleosome level, and a high ICAM-1 level were independent prognostic factors for the primary composite endpoint in incident HD patients.

**Table 2:** Predictors of long-term composite outcomes.

Variables	Unit	Composite outcomes			
		Univariate		Multivariate†	
		HR (95% CI)	P	HR (95% CI)	p
Age	> 65 years	1.94 (0.13–3.29)	0.017	1.92 (1.09–3.41)	0.025
Gender	Male vs. female	0.75 (0.46–1.30)	0.337	–	–
SBP	Per 10 mmHg	1. 01 (0.99–1.03)	0.076	–	
Diabetes	Presence	1.00 (0.59–1.69)	0.998	–	–
Nucleosome, Q4	Q 1–3	2.79 (1.72–4.53)	< 0.001	2.86 (1.69–4.83)	< 0.001
ICAM-1	Per 1 increase	1.002 (1.001–1.003)	0.003	1.001 (1.000–1.003)	0.033

^†^Adjusted for age, gender, blood pressure, diabetes, nucleosome and ICAM-1.

### Differentiation of HL-60 cells

**Figure 3:**
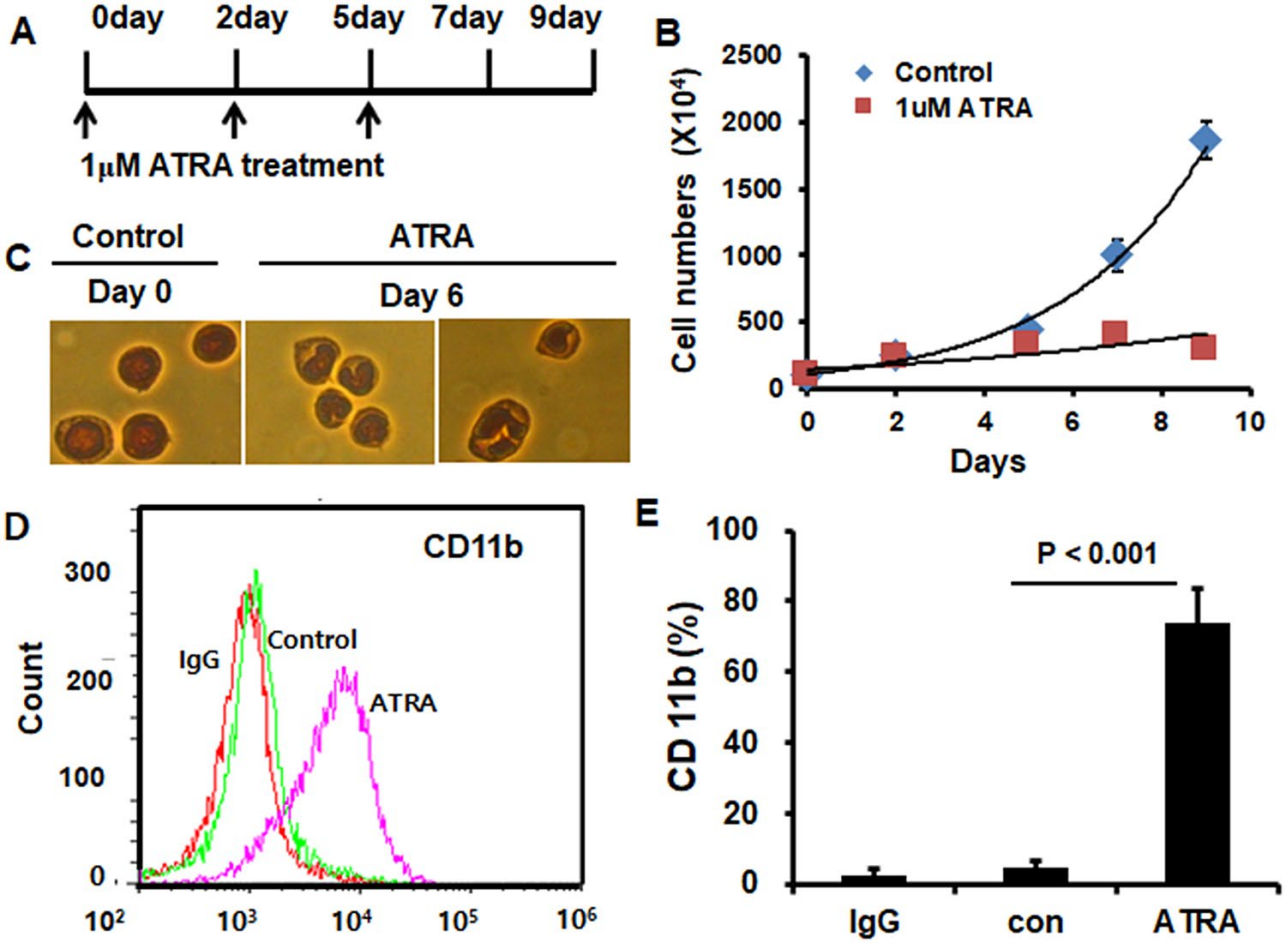
HL-60 cells differentiated to neutrophil-like cells by all–trans-retinoic acid (ATRA). (**A**,**B**) Growth curve. HL60 cells (5 × 10^5^/mL) were seeded into a T-75 flask and treated with 0 or 1 μM ATRA once every 2 to 3 days for 5 days. Cells not stained by trypan blue were counted for 9 days. (**C**) Nuclear morphology of HL60 cells. HL60 cells were treated with 0 or 1 μM ATRA for 6 days and stained with Wright-Giemsa. At day 0, the nuclear morphology of dHL60 cells was ovate (left) but showed a dent (center) or lobular shape at 6 days (right) (magnification, ×400). (**D**,**E**) FACS analysis of CD11b expression in dHL60 cells. HL60 cells were differentiated by 0 or 1 μM ATRA for 6 days and stained with an anti-CD11b-FITC antibody. IgG was used as the negative control. Expression of CD11b (left). Percentage of total cells expressing CD11b (right). Independent sample t-test was used to find the difference.

Human promyelocytic HL-60 cells can be induced to differentiate to neutrophil-like cells (dHL-60) in response to a variety of chemical stimuli. As shown in Fig. 3, three applications of 1 μM ATRA on days 0, 2, and 5 day (Fig. 3A) stopped the proliferation of HL-60 cells by day 5 (Fig. 3B). However, DMSO (0.01%) did not affect the differentiation of HL-60 cells as determined by cell growth curve (data not shown). Cells cultured with ATRA exhibited morphological changes characteristic of mature polymorphonuclear cells (PMNs). By day 6, cells of mature neutrophil morphology accounted for > 75% of total cells (Fig. 3C). FACS confirmed the differentiation of HL-60 cells into neutrophil-like dHL60 cells with increased expression of CD11b (Fig. 3D,E).

### Induction of NETs in dHL-60 by uremic serum

**Figure 4:**
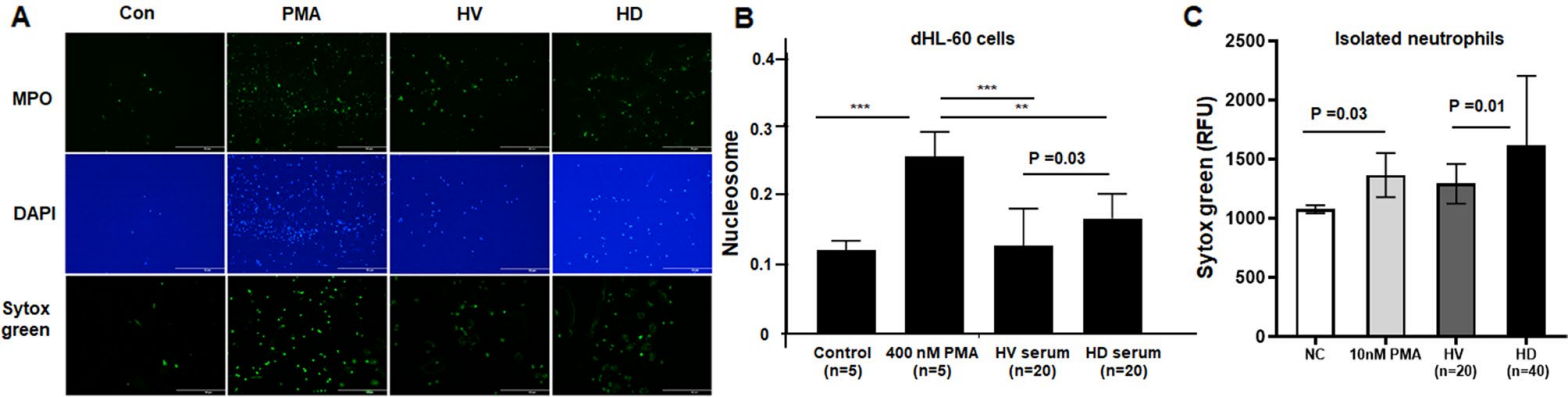
Induction of NETs by dHL60 cells. (**A**) Detection of NETs by immunofluorescence. HL60 cells were differentiated for 6 days and treated with 0 or 400 nM PMA, HV serum, or HD serum for 17 h. NETs were visualized by co-staining with myeloperoxidase (MPO) and DAPI, or by staining with Sytox Green (magnification, ×100). (**B**) Quantification of cell-free nucleosomes with dHL-60. HL-60 cells were differentiated for 6 days and treated with 0, 400 nM PMA, HV serum, or HD serum for 17 h. DNA released from the nucleus was digested by nuclease. More nucleosomes were released in HD serum than in HV serum. (**C**) The release of NETs from fresh isolated neutrophils. Similar to the results with dHL-60 cells, uremic serum induced significantly increased NETs compared to control serum. Results are means ± SEM, Student’s *t*-test.

Stimulation of dHL-60 cells with PMA significantly increased NETosis and the supernatant MPO-DNA and nucleosome levels compared with the control (p < 0.001) (Fig. 4A). Next, we treated dHL-60 cells with serum from HD patients (n = 40) and HV (n = 20) and evaluated NET formation. As shown in Fig. 4A, uremic serum-stimulated dHL-60 cells released more NETs than dHL-60 cells stimulated with HV-derived serum. The nucleosome level in dHL-60 cell supernatants was also significantly increased by uremic HD serum compared to the control (p < 0.001) and HV sera (p = 0.03) (0.16 for HD serum vs. 0.12 for HV serum) (Fig. 4B). However, the level was significantly lower than that induced by the positive control activator, 400 nM PMA (p < 0.01). To confirm this finding, we also repeated the test with HV-derived neutrophils too. Very similarly to the findings with dHL-60 cells, uremic serum produced significantly higher levels of NETs from HV-derived neutrophils compared to control serum (p = 0.01) (Fig. 4C). Together with our in vivo data, these results suggest that neutrophil activation is increased in the uremic environment, promoting NET formation.

### NETosis induced by uremic serum increases EC damage

**Figure 5:**
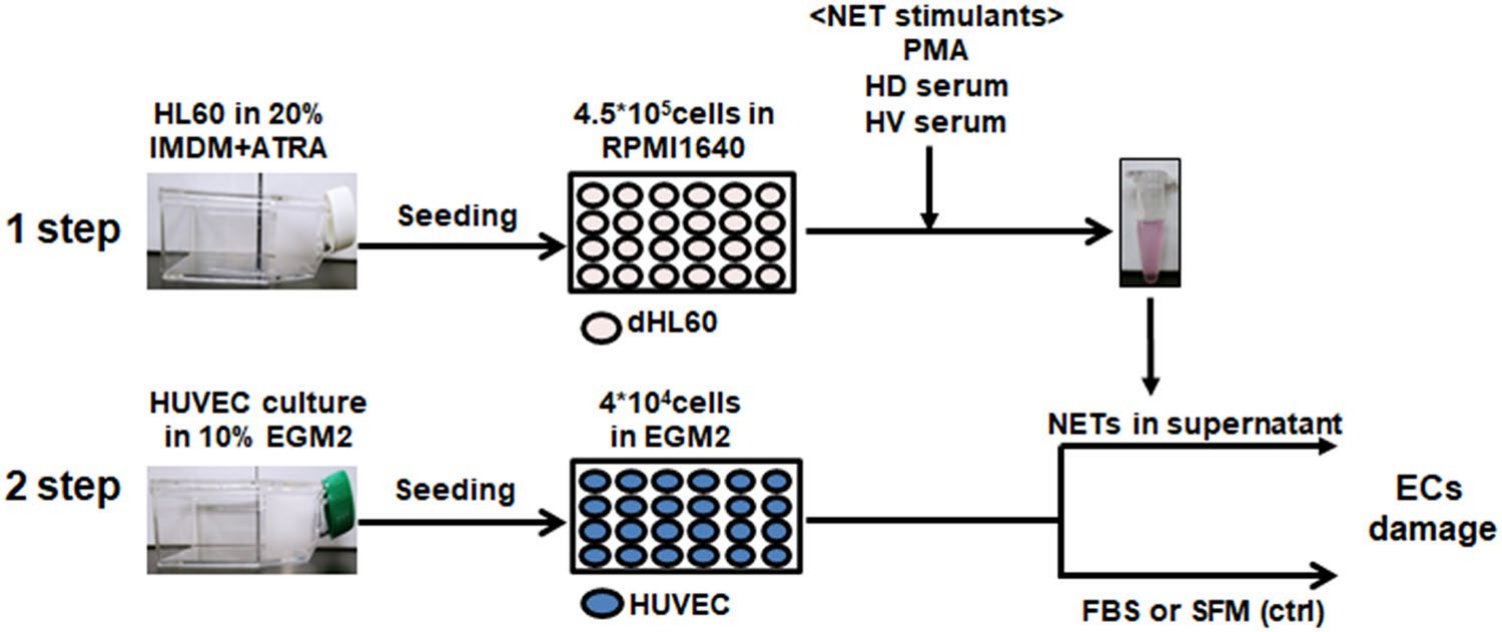
Schematic diagram of the experimental procedure. HL-60 cells were seeded into T75 flasks at 5 × 10^5^ cells/mL and cultured for 6 days in 20% IMDM with 1 nM ATRA. Differentiated HL-60 cells were seeded into 24-well plates at 4.5 × 10^5^ per well and cultured in RPMI1640 with 1% BSA. PMA, HV serum and HD serum were applied to dHL-60 cells for 17 h and nuclease was added for 1 h. Cell free–NETs in supernatants were transferred to a new e-tube containing EDTA. HUVEC cells were cultured in 10% EGM2 and 4 × 10^4^ were seeded into 24-well plates. The day before the experiment, the culture medium was replaced with serum-free medium (SFM), 10% FBS, or 10–20% of NET supernatant. Cells were incubated for 20 h and used for analysis.

**Figure 6:**
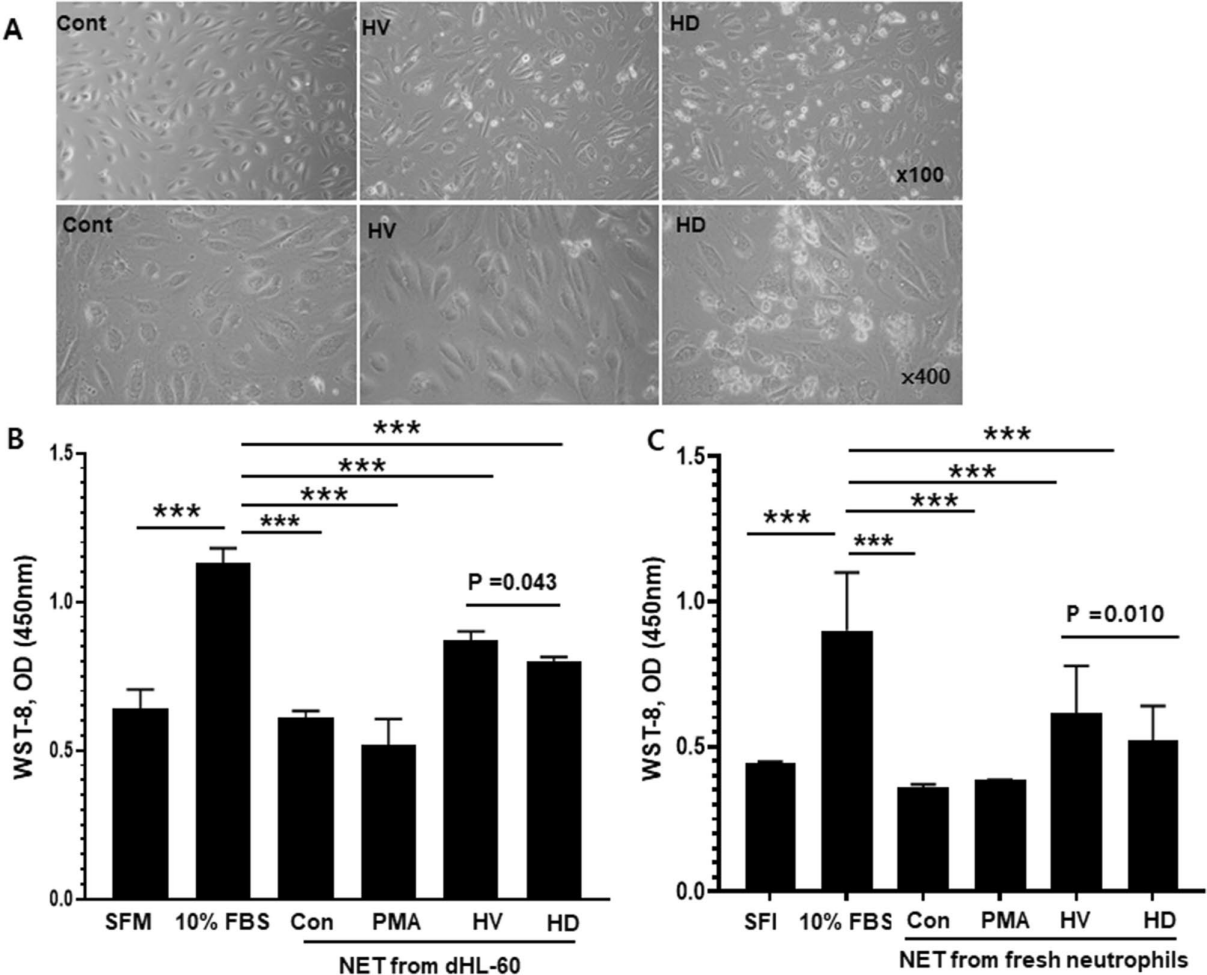
Damage to endothelial cells by NETs. To assess HUVEC damage, control (0 nM PMA), 400 nM PMA, HV serum, and HD serum were applied to dHL-60 cells for 17 h. Supernatants (10%) were added to HUVECs and incubated for 20 h. Serum-free medium (SFM) and 10% FBS were used as controls. (**A**) Morphology of HUVECs. In HV and HD NETs, cells became elongated compared to the control, and many dead floating cells were observed (magnification, upper panels, ×100; lower panels, ×400). (**B**) Effect of NETs from dHL-60 cells on HUVEC viability was tested by WST-8 assay. HUVEC viability was higher in HV serum-induced NETs than in uremic serum-induced NETs and SFM, and lower than those in 10% FBS. (**C**) Repeat experiments with isolated neutrophils from blood donor. Results are means ± SEM, Student’s *t*-test, ***p < 0.001.

To test the hypothesis that increased NETosis in uremic serum induces endothelial damage, we evaluated HUVEC viability, apoptosis, and ICAM-1 expression in response to uremic serum- induced NETs (Fig. 5). First, we treated dHL-60 cells with 10% uremic or normal serum and PMA to induce NET release. Cells were incubated at 37 ℃ for 17 h, treated with nuclease for 1 h, and supernatants collected by centrifugation and applied to HUVECs. SFM and 10% FBS were used as the controls. Representative phase-contrast micrographs of HUVEC morphology are shown in Fig. 6. Treatment with uremic serum for 20 h resulted in a significant proportion of HUVECs losing their “cobblestone” appearance, becoming elongated and spindle-like compared to the control (Fig. 6A). Consistent with this, a WST-8 test showed that the number of viable HUVECs was significantly decreased by SFM or PMA-induced NETs compared to 10% FBS. Incubation of HUVECs treated with uremic serum-induced NETs showed significantly decreased viability (by 18.2%; p = 0.043) compared with those treated with HV serum-induced NETs (Fig. 6B). In a repeat experiment with HV-derived neutrophils, similar findings were observed, too (Fig. 6C).

**Figure 7:**
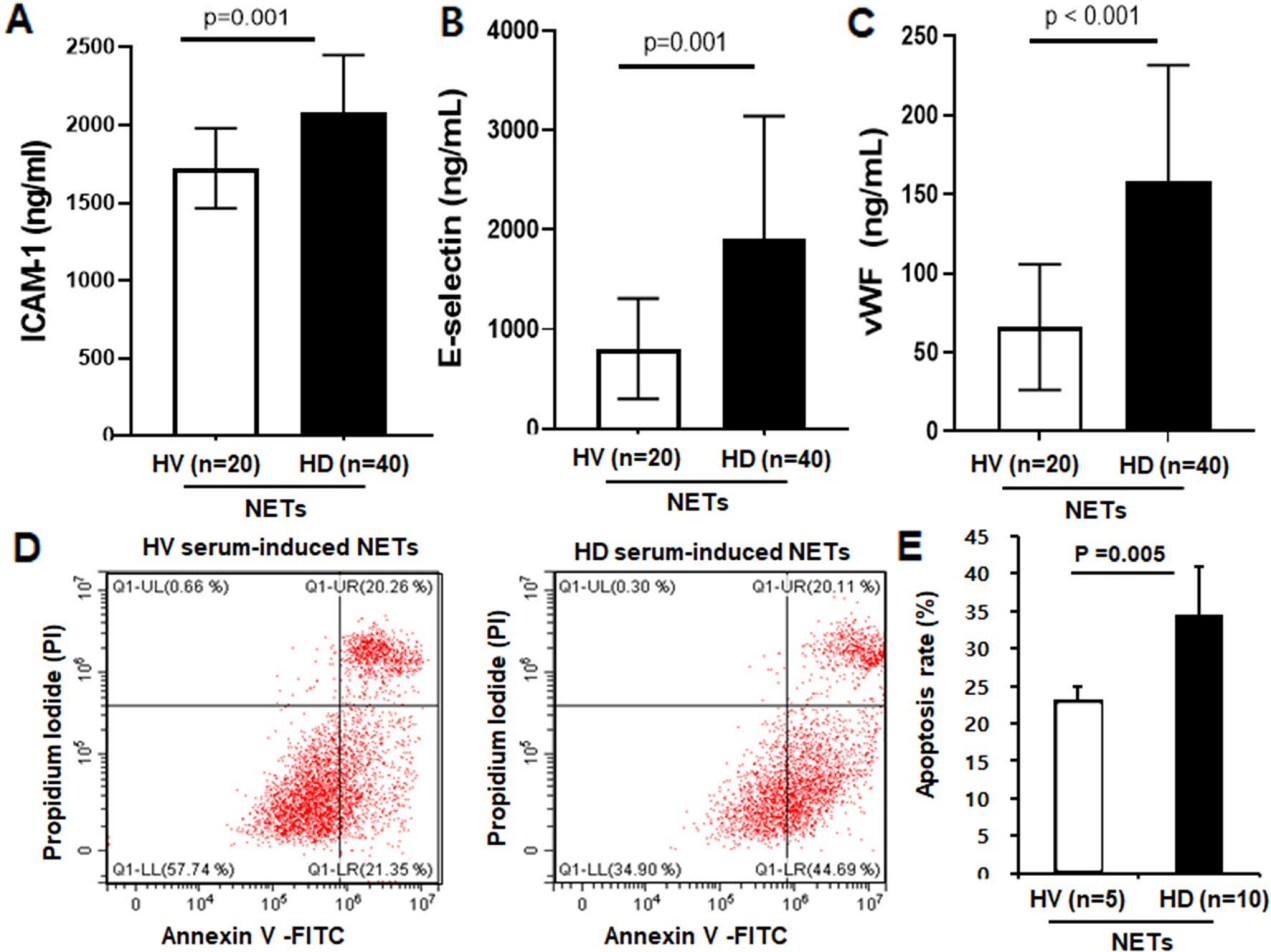
NETs promote apoptosis of endothelial cells. (**A**–**C**) Intercellular adhesion molecule (ICAM)-1, E-selectin and von Willebrand factor (VWF) levels in HUVEC supernatants as determined by ELISA. The levels were higher with HD serum-induced NETs than for HV serum-induced NETs. Results are means ± SEM, Student’s *t*-test, ***p < 0.001. To find the apoptosis in HUVEC by NETs, HV or HD serum were applied to dHL60 cells for 17 h. Then, the supernatants (20%) were applied to HUVECs for 20 h, and the cells were fixed and stained with Annexin V and propidium iodide. (**D**,**E**) FACS analysis. Representative flow plot of the distribution of early apoptosis (Annexin V positive and propidium iodide negative). Percentage of total cells counted. HUVEC Apoptosis was greater for HD serum-induced NETs than HV serum-induced NETs. Results are means ± SEM, Student’s *t*-test.

Furthermore, the ICAM-1, E-selectin and vWF levels in HUVEC supernatant were significantly higher when treated with uremic serum-induced NETs than normal serum-induced NETs (Fig. 7A–C). Finally, the percentage of early apoptotic cells was analyzed by flow cytometry. The apoptosis rate of HUVECs stimulated by HV serum-induced and uremic serum-induced NETs were 25% and 34.6%, respectively (Fig. 7D,E). The increased NETosis induced by uremic serum might decrease endothelial viability and increase cell damage and apoptosis. Therefore, the endothelial dysfunction in a uremic milieu depends on excessive NET production and immune dysregulation.

## Discussion

We report that uremic serum from HD patients induces excessive NET production by neutrophils, leading to decreased EC viability and increased EC apoptosis. Therefore, dysregulated innate immunity in a uremic milieu may play a key role in the vascular inflammatory response. The high mortality and CVD complication rates of ESRD patients can be explained in part by dysregulated neutrophils, excessive NET formation, and EC dysfunction.

Preservation of the structure and functions of ECs is fundamental for vascular health. Endothelial dysfunction, the basis of atherosclerosis, is evident at an early stage in CKD, and its prevalence increases as patients progress toward ESRD. Chronic exposure to various uremic toxins exerts deleterious effects on vascular ECs, damaging their monolayer structure and triggering a proinflammatory and a prothrombotic phenotype. These changes contribute to the pathogenesis of accelerated atherosclerosis and CVD independently of hypertension. Uremic toxins from CKD patients induce loss of cell–cell junctions and increase endothelial permeability^28^. In addition, exposure of ECs to uremic serum in vitro results in endothelial injury and inflammation, characterized by an increase in MCP-1 expression. These alterations activate the vascular repair process by increasing the expression of vascular endothelial growth factor and stromal cell-derived factor 1^29^.

We hypothesized that immune dysregulation, particularly neutrophil dysregulation, may be actively involved in endothelial dysfunction since uremic toxins can enhance neutrophil activation, ROS formation and excessive NET release^30,31^. This dysregulated response could impair programmed cell death of endothelial cells, leading to chronic sterile inflammation in vascular walls. In addition, NETosis might disrupt the highly regulated interactions between neutrophils and ECs, causing adhesion of neutrophil to inflamed endothelium and overactivation. Indeed, plasma from CAD patients with eroded plaques has an elevated MPO level, produced primarily by neutrophils, as compared to those with ruptured lesions^32^. Co-culture of neutrophils with ECs induces endothelial injury and hypochlorous acid, a major product of MPO, and can trigger endothelial cell apoptosis and generation of tissue factors^33^.

We first compared NETosis in HV and HD patients with and without adverse vascular events. Plasma nucleosome and MPO-DNA levels, an established marker of in vivo NETs, as well as circulating neutrophil counts were significantly higher in HD patients compared to HVs, and they were much higher in HD patients with adverse vascular events compared to those without. Also, in vivo NETs were strongly associated with the plasma ICAM-1 level. These correlations suggest that a chronic uremic condition promotes excessive NETosis and EC damage. Moreover, we also found that the increased nucleosome and ICAM-1 levels were independent predictors for adverse vascular outcome; those patients with particularly high nucleosome levels (Q4) and increased ICAM-1 level showed the lowest 5-year survival rate. Based on these findings, we can suggest that increased neutrophil activation with higher NETs release may be one of the main causes of endothelial dysfunction in CKD, resulting in increased risk of adverse CV outcomes. Supporting our data, there is a body of evidence indicating that elevated levels of circulating DNA and chromatin released from activated neutrophils are independently associated with severe coronary atherosclerosis and thrombosis, facilitating progression of vascular complications^34^.

Next, we investigated the direct effect of uremic serum on EC damage and injury. Uremic toxins could mediate endothelial dysfunction and vascular inflammation, and thus implicated in endothelial dysfunction of CKD^6,9^. There have been potentially conflicting data reported on the effect of uremic serum on endothelial dysfunction. Andrea et al. found that uremic serum induced a slight decrease in cell proliferation and viability and increased apoptosis compared to normal serum^10^. However, uremic serum induced apoptosis in aging, but not in young, HUVECs^35^. The discrepant results may be explained by use of different concentrations and durations of exposure to uremic serum. However, the mechanisms by which uremic serum influences ECs, especially the early responses of EC injuries, are unclear. We did not evaluate the effects of the myriad individual uremic toxins but, instead, used uremic serum. Indeed, uremic syndrome is attributed to progressive retention of multiple uremic compounds as well as chronic inflammation and immune dysregulation.

We hypothesized that dysregulated innate immunity, particularly overactivation of neutrophils and the resultant increase in NETosis, are implicated in the above-mentioned mechanism. Uremic serum from HD patients significantly increased NET formation both in isolated neutrophils and dHL-60 cells compared to normal serum. Subsequently, the NETosis induced by uremic serum decreased HUVEC viability and increased apoptosis. Similarly, the markers of endothelial damage were all increased by stimulation with NETs released by uremic serum compared to normal serum.

Neutrophils regulate endothelial barrier function via adhesion-dependent and secretion-dependent mechanisms. NETs are involved in secretion-dependent neutrophil-EC interactions. Uremia is characterized by chronic inflammation and increased oxidative stress, which in turn cause neutrophil activation and excessive NET generation. Indeed, one subtype of NETosis, “suicidal” NETosis, is characterized by strong activation of nicotinamide adenine dinucleotide phosphate (NADPH) oxidase by PMA or other microbial pathogens in an ROS-dependent manner^36,37^. ROS induce gradual separation and loss of the nuclear membrane with extracellular release of chromatin through membrane pores, promoting NETosis^25,38^. This disrupts EC junctions, induces glycocalyx degradation, focal adhesion reorganization, and cytoskeletal contraction, leading to intercellular gap formation and increased para-endothelial permeability. In vitro, NETs increase the flux of albumin and 10 kDa dextran across EC monolayers. Neutralizing NET components by DNase 1, or inhibition of NET formation by a PAD2/4 inhibitor or PAD4 gene deletion, reduces lung vascular permeability in murine models of transfusion-related acute lung injury.

Overall, our results demonstrated that uremic serum accelerates EC damage and endothelial dysfunction by triggering excessive NETosis by neutrophils, possibly by increasing oxidative stress. Uremic serum increased EC apoptosis and ICAM-1 expression, and decreased HUVEC viability. Control of NETs shows promise for preventing endothelial damage and improving the clinical outcomes of CKD patients, emphasizing the need for immunomodulatory therapies.

## Materials and methods

We first measured markers of in vivo NETs and endothelial damage and evaluated their relationship and prognostic significance for long-term vascular complications in incident ESRD patients. In parallel, the response of ECs to NETs induced by uremic serum was evaluated in vitro.

### Study population and blood sampling

Samples were collected from 51 healthy volunteers (HV) and 201 incident HD patients who initiated HD between January 2013 and December 2017. This study was approved by Hallym University Sacred Heart Hospital Institutional Review Board and conducted in accordance with the Declaration of Helsinki. Informed consent was obtained from the HD patients and HVs. Baseline demographic data, including age, sex, comorbidities, history of CVD, and clinical data regarding the underlying cause of ESRD were obtained. Venous sampling was performed immediately prior to each patient’s first HD session. Each sample was collected using EDTA tubes (367835, BD) for plasma, and Serum Separator Clot Activator tubes (456073, BD) for serum. Plasma was separated by centrifuging at 1500*g* for 10 min at 4 °C and serum was incubated for 20 to 30 min at room temperature and centrifuged as for plasma. Isolated plasma and serum were aliquoted and stored at − 70 °C until analyzed. Biochemical analyses of white blood cells (WBCs), neutrophils, lymphocytes, and the levels of hemoglobin, serum albumin, urea nitrogen, and creatinine were performed. Neutrophil to lymphocyte (N/L) ratios were also calculated.

### Measurement of markers of in vivo NETs and endothelial damage

To quantify in vivo NETs, we measured the circulating nucleosome (histone-DNA) and myeloperoxidase-DNA (MPO-DNA) levels (Cell Death Detection ELISA Plus Kit, Roche Diagnostics) in plasma as in our previous paper. To assess endothelial damage, a commercially available ELISA kit was used for plasma ICAM (Intercellular Adhesion Molecule) -1 (DY720, R&D Systems) and E-Selectin and von Willebrand factor (vWF) (Ray Biotech, USA). We next evaluated their associations and prognostic roles for long-term adverse vascular outcomes.

### Vascular outcomes

The primary composite endpoints of this study were all-cause mortality and vascular access failure. We used vascular access failure as an endpoint because vascular access complications depend on endothelial damage. Therefore, the end of follow-up was determined by death from all-causes or permanent abandonment of HD vascular access. For patients without events, the last in-patient contact was recorded until December 2020. In cases of both events, the last date was used as the date of the first event.

### In vitro experiments

#### HL-60 cell culture and differentiation

HL-60 cells (CCL-240, ATCC) were maintained in Iscove’s modified Dulbecco’s medium (12440053, IMDM, Thermo Fisher Scientific) with 20% fetal bovine serum (FBS; 97068-85, VWR, Vienna, Austria). To differentiate HL-60 cells into neutrophil-like cells, the cells were incubated in culture medium containing 1 μM all–trans-retinoic acid (ATRA; R2625, Sigma-Aldrich) dissolved in dimethyl sulfoxide (DMSO; D2650, Sigma-Aldrich). Cells were grown at 37 °C in a 5% CO_2_ incubator in T-75 culture flasks (70375, SPL Life Science, Pocheon, South Korea). The medium was changed once every 2 to 3 days for 9 days and contained 1% penicillin–streptomycin (LS202, Welgene, Daegu, Korea) and 0.1% mycoplasma removal agent (093050044, MP Biomedicals, lllkirch, France).

To confirm that the HL-60 cells were adequately differentiated into multiloculated PMNs, Wright-Giemsa staining was performed. The differentiated HL-60 cells (dHL-60) were smeared on a clean slide glass using a cover glass, air-dried, fixed in 100% methanol for 5 min, and stained for 5 min with a Wright-Giemsa Staining Kit (K1438, Biovision, Milpitas, CA). Stained cells were rinsed with deionized water (DW) and then with PBS (pH 6.8), dehydrated with xylene and mounted (H-1000, Vectashield, Burlingame, CA). For quantification, dHL60 cells were centrifuged, washed in PBS, resuspended in staining buffer (2% BSA in PBS), and Fc blocker (564220, BD) was added; the cells were then incubated for 10 min. Next, cells were reacted with a FITC-IgG (555748, BD) or FITC-CD 11b (562793, BD) antibody for 20 min in a dark room. Stained cells were centrifuged, washed in PBS, and fixed in 1% paraformaldehyde. Finally, CD11b expression in dHL-60 cells was measured.

### NET induction by dHL-60 cells

dHL-60 cells were cultured in RPMI1640 containing 1% BSA, 1% antibiotics, and 10 mM HEPES (HO887, Sigma-Aldrich). To stimulate NET formation in dHL-60 cells, 100, 200, and 400 nM phorbol myristate acetate (PMA; 601010, Cayman, Ann Arbor, MI) or serum from HD patients was used. As a control, serum from HVs was used. HL-60 cells differentiated for 6 days by ATRA were seeded at 5 × 10^5^ cells in a 24-well plate and incubated for 17 h. Next, cells were treated with 150 U/mL S7 nuclease (601010, Cayman) for 1 h and the supernatant was transferred to a microtube (MCT-150-C, AXYGEN, Corning, NY). Nuclease was inactivated by adding 10 mM EDTA to the supernatant and centrifugation at 500*g* for 5 min. The supernatant was transferred to a new microtube and stored at – 20 °C until analysis.

### Immunofluorescence staining of NETs

dHL-60 cells (3 × 10^5^/well) were seeded in a four-well slide chamber (30504, SPL Life Science) and treated with 400 μM PMA for 17 h to induce NETosis. Formaldehyde-fixed cells were washed with PBS and blocked with 3% BSA for 30 min. The blocking solution was removed, and MPO (ab25989, Abcam, 1:250) was added overnight at 4 °C. An IgG primary antibody was used as the negative control. The next day, antibodies were removed by washing with PBS and incubated for 2 h with an Alexa 488 goat anti-mouse antibody (A11001, Invitrogen). The cells were mounted in a solution containing DAPI (H1200, Vector Laboratories, Burlingame, CA). For Sytox Green staining, PMA-stimulated cells were washed with HBSS (14025-092, Thermo Fisher Scientific) and treated with 5 μM Sytox Green (S7020, Thermo Fisher Scientific) for 20 min in the dark. After washing with HBSS, cells were mounted. Cells were observed under a fluorescence microscope (Eclipse Ni, Nikon, Tokyo, Japan).

### Isolation of neutrophils

In addition to the in vitro test with dHL-60 cells, we also tested our hypothesis with HV-derived fresh neutrophils, too. Venous blood was taken from 5 healthy donors into EDTA-tube and PMN were isolated by Ficoll density gradient centrifugation. The 8 × 10^5^ cells were seeded to 96 well plate and serum from HV (n = 20) or HD patients (n = 40) were treated and incubated 3 h. And then neuclease were treated and incubated for 10 min. 5 mM EDTA for the inhibition of the enzyme activity were treated. Plate was centrifuged and the supernatant were collected for Sytox green assay and cyto-toxicity test to HUVEC.

### Sytox green assay

100ul supernatant and 100ul of Sytox green (S7020, invitrogen) were add to 96well plate and incubated for 5 min. The level of stained DNA was measured at 500–550 nm wave lengths.

### HUVEC stimulation with NETs

To evaluate whether NETs induced from dHL-60 cells cause endothelial injury, human umbilical vein endothelial cells (HUVECs; CRL-1730, ATCC) were cultured in endothelial growth medium (EGM)-2 (CC-3162, Lonza, Basel, Switzerland) and the medium was changed once per 2 to 3 days. Other conditions were as for dHL-60 cells. HUVECs were seeded at 4 × 10^4^ cells in a 24-well plate in 1% EGM2 culture medium. The next day, the medium was exchanged for serum free-EGM2 and dHL-60 supernatant, in which NETs were induced by serum from HD patients and HVs and incubated for 20 h. HUVEC supernatant was centrifuged at 500*g* for 5 min, transferred to a new 1.5 mL tube, and 10 mM EDTA was added. HUVEC supernatant was stored at – 20 °C until analysis.

### Damage to HUVECs

HUVEC viability was assayed by adding Cell Counting Kit-8 (CCK-8, CK04, Dojindo, Rockville, MD) reagent and incubation for 4 h. HUVECs stimulated with serum-free medium (SFM) and 10% FBS were used as the controls. To evaluate EC apoptosis, HUVECs were seeded at 4 × 10^4^ cells and stimulated with 20% dHL-60 supernatant, which had been stimulated with uremic or normal serum for 20 h. Next, cells were washed with PBS and trypsinized. Detached cells were centrifuged, washed in PBS, stained using an Annexin-V-FLUOS Staining Kit (11 858 777 001, Roche), and 1 × 10^4^ cells were subjected to flow cytometry (CytoFLEX, Beckman Coulter).

### Statistical analysis

Variables with normal distributions are expressed as means ± standard deviation (SD) and Kolmogorov–Smirnov tests were used to analyze the normality of distribution. Categorical variables are expressed as percentages and were compared by chi-squared test. An independent sample t-test was used to find the difference among the groups based on continuous values. Pearson’s correlation coefficient was calculated for circulating nucleosomes, MPO-DNA, ICAM-1, and various biochemical factors and comorbidities. Cumulative survival curves were derived using the Kaplan–Meier method, and survival curves were compared by log-rank test. The patients in the lower three quartiles (Q1–3) of circulating nucleosomes, treated as the reference, were compared with those in the highest quartile (Q4). In vitro studies were conducted in duplicate or triplicate and repeated independently at least twice. Data are graphed as means with standard deviation (SD). Statistical evaluation was conducted by one-way analysis of variance (ANOVA) or unpaired two-tailed Student’s *t*-test (*p < 0.05; **p < 0.01; ***p < 0.001).

## Supplementary Information

Supplementary Figure S1.

Supplementary Figure S2.

## Data Availability

The datasets generated during and/or analysed during the current study are available from the corresponding author on reasonable request.
