# Supplementary figures and images for: Uremic serum damages endothelium by provoking excessive neutrophil extracellular trap formation
Source: Sci Rep. 2021 Nov 2;11:21439. doi: 10.1038/s41598-021-00863-w (PMC8563801; doi:10.1038/s41598-021-00863-w)

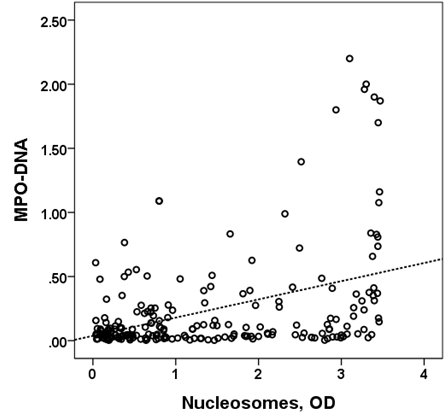

Supplement: Supplementary file 1 — Supplementary Figure S1. [file 41598_2021_863_MOESM1_ESM.tif]

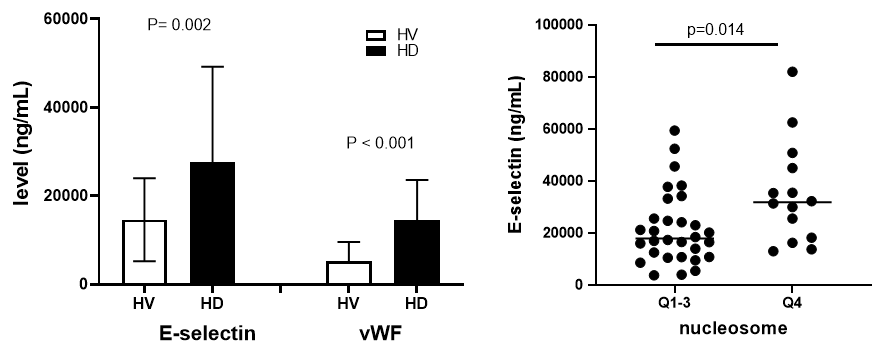

Supplement: Supplementary file 2 — Supplementary Figure S2. [file 41598_2021_863_MOESM2_ESM.tif]
